# Supplementary material for: Preterm birth and secondhand smoking during pregnancy: A case–control study from Vietnam
Source: PLoS One. 2020 Oct 7;15(10):e0240289. doi: 10.1371/journal.pone.0240289 (PMC7540896; doi:10.1371/journal.pone.0240289)
Supplement: S1 Questionnaire — (DOCX) [file pone.0240289.s001.docx]

**SHS questionnaire Vietnamese version**

**BẢNG PHỎNG VẤN HÚT THUỐC LÁ THỤ ĐỘNG**

Nghiên cứu này giúp chúng tôi xác định ảnh hưởng của khói thuốc lá trên sức khỏe của trẻ sơ sinh. Phỏng vấn này có tính cách tự nguyện và kéo dài khoảng 10 phút. Chị/Bà có đồng ý tham gia nghiên cứu này?. Chữ ký: ________ Ngày: ________

Họ tên: ---------------------------------------------- Số BA: -------------------------

Cân nặng (kg): ------------------------------------ Chiều cao (cm):---------------

Chị/Bà bao nhiêu tuổi? ________

Trình độ học vấn cao nhất đã hoàn tất

1. Tiểu học (lớp 1-5) -----------------------------------------------------------
2. Trung học (lớp 6 - 12) ------------------------------------------------------
3. Cao đẳng/ Đại học -----------------------------------------------------------

Chị/Bà làm nghề gì?

1. Nội trợ -------------------------------------------------------------------------
2. Nông dân ----------------------------------------------------------------------
3. Công chức Nhà nước -----------------------------------------------------
4. Nhân viên tư nhân --------- ------------------------------------------------
5. Học sinh, sinh viên ----------------------------------------------------------
6. Nghề tự do --------------------------------------------------------------------

c

1. Thất nghiệp -------------------------------------------------------------------

**Hút thuốc lá thụ động**

Có *ai* hút thuốc lá trong nhà Chị/Bà? Hút mỗi ngày, mỗi tuần, mỗi tháng hoặc không bao giờ hút ?

Không bao giờ Có

Nếu có,

Có bao nhiêu người hút thuốc lá trong nhà trong vòng 9 tháng nay ?---------------

Mỗi người hút bao nhiêu điếu thuốc lá trung bình mỗi ngày ? ---------------------------

Thành viên 1 (Chồng): 1-5 6-10 10+

Thành viên 2: 1-5 6-10 10+

Thành viên 3: 1-5 6-10 10+

**Tiền sử sản khoa**

Trong thởi gian mang thai lẩn này, Chị/Bà có đi khám thai tại phòng Chăm sóc sức khỏe sinh sản?

Không Có Số lần : -----------------

Trong lần mang thai này, Chị/Bà có bị bệnh gì không ?

v

1. Đái tháo đường thai kỳ -----------------------------------------------------

v

1. Tăng huyết áp ----------------------------------------------------------------

v

1. Chảy máy âm đạo trong thời kỳ mang thai ---------------------------

v

1. Tiền sản giật hoặc sản giật -----------------------------------------------

- v

1. Không bị bệnh gì -------------------------------------------------------------

Chị/Bà sinh mấy lần rồi (≥22 tuần thai)? --------------------

Số con còn sống: -----------------

Chị/Bà có tiền sử bị sinh non không? Không Có

v

Chị/Bà có tiền sử bị hư thai không? Không Có

**SHS questionnaire English version**

**SECONDHAND SMOKING QUESTIONNAIRE**

This research study will help us determine the effects of secondhand smoke on your child health. The survey is voluntary and will take about 10 minutes. Do you agree to participate in this survey. Please, signed: ----------------- date:---------------- (dd/mm/yy)

Full name: ----------------------------------------- ID number: ------------------

Body weight (kg): ------------------------------- Height (cm):------------------

What is your age? ________

What is the highest level of education you have completed

1. Primary school (grade 1-5) completed----------------------------------
2. Secondary school (grade 6-12 ) completed------------------------------------
3. College/ University completed---------------------------------------------

What is your occupation?

1. Housewife-----------------------------------------------------------------------
2. Farmer---------------------------------------------------------------------------
3. Government employee -----------------------------------------------------
4. Non-government employee ------------------------------------------------
5. Student --------------------------------------------------------------------------
6. Self-employed -----------------------------------------------------------------
7. Unemployed -------------------------------------------------------------------

**Secondhand Smoking**

How often does *anyone* smoke inside your home? Would you say daily, weekly, monthly, or never?

Never Yes

If Yes

How many members smoke inside your home in the last 9 months?-------------------

On average, how many cigarettes do they currently smoke each day?-----------------

Member 1 (husband): 1-5 6-10 10+

Member 2: 1-5 6-10 10+

Member 3: 1-5 6-10 10+

**Obstetric History**

During your most recent pregnancy, did you attend the antenatal care unit?

No Yes How many times: -----------------

During your most recent pregnancy, did you have any of the following

health conditions?

1. Gestational diabetes --------------------------------------------------
2. High blood pressure ---------------------------------------------------
3. Vaginal bleeding during pregnancy --------------------------------
4. Pre-eclampsia or eclampsia -----------------------------------------
5. Not at all ------------------------------------------------------------------

How many times you give births ( ≥22 weeks)?----------

Number of alive children-----------

Do you have a history of premature birth? No Yes

Do you have a history of miscarriage? No Yes
