# Supplementary material for: Preterm birth and secondhand smoking during pregnancy: A case–control study from Vietnam
Source: PLoS One. 2020 Oct 7;15(10):e0240289. doi: 10.1371/journal.pone.0240289 (PMC7540896; doi:10.1371/journal.pone.0240289)
Supplement: Attachment — Submitted filename: RESPONSE TO REVIEWERS.docx [file pone.0240289.s003.docx]

Response to Reviewer’s

1. Please ensure that your manuscript meets PLOS ONE's style requirements, including those for file naming. The PLOS ONE style templates can be found at

<https://journals.plos.org/plosone/s/file?id=wjVg/PLOSOne_formatting_sample_main_body.pdf> and

<https://journals.plos.org/plosone/s/file?id=ba62/PLOSOne_formatting_sample_title_authors_affiliations.pdf>

it’s done in Revised Manuscript

2. Please address the following:
- Please include additional information regarding the survey or questionnaire used in the study and ensure that you have provided sufficient details that others could replicate the analyses. For instance, if you developed a questionnaire as part of this study and it is not under a copyright more restrictive than CC-BY, please include a copy, in both the original language and English, as Supporting Information. Please include further details of the development and validation of this questionnaire, including any pre-testing that took place.
- Please refrain from stating p values as 0.000, either report the exact value or employ the format p<0.001.
- Please provide additional details regarding participant consent. In the ethics statement in the Methods and online submission information, please ensure that you have specified what type of consent you obtained (for instance, written or verbal, and if verbal, how it was documented and witnessed).

The questionnaires in original language (Vietnamese) and English are available in the Supporting Information (S1 questionnaire.docx)

We included the details of the development and validation of the questionnaires in the Methods of Revised manuscript

We replaced p values as 0.000 with the format <0.001

We provided more details regarding participant consent and signed before

attending the review in Ethical issues of the Revised manuscript

3. Thank you for stating the following financial disclosure:

"The funders had no role in study design, data collection and analysis, decision to publish, or preparation of the manuscript."

At this time, please address the following queries:

a. Please clarify the sources of funding (financial or material support) for your study. List the grants or organizations that supported your study, including funding received from your institution.
b. State what role the funders took in the study. If the funders had no role in your study, please state: “The funders had no role in study design, data collection and analysis, decision to publish, or preparation of the manuscript.”
c. If any authors received a salary from any of your funders, please state which authors and which funders.
d. If you did not receive any funding for this study, please state: “The authors received no specific funding for this work.”

Please include your amended statements within your cover letter; we will change the online submission form on your behalf.

We declared “The authors received no specific funding for this work.” in cover letter and in the Revised manuscript

4. Please include your tables as part of your main manuscript and remove the individual files. Please note that supplementary tables (should remain/ be uploaded) as separate "supporting information" files.

We included the tables in the revised manuscript

**Comments to the Author**

1. Is the manuscript technically sound, and do the data support the conclusions?

The manuscript must describe a technically sound piece of scientific research with data that supports the conclusions. Experiments must have been conducted rigorously, with appropriate controls, replication, and sample sizes. The conclusions must be drawn appropriately based on the data presented.

Reviewer #1: Partly

2. Has the statistical analysis been performed appropriately and rigorously?

Reviewer #1: Yes

3. Have the authors made all data underlying the findings in their manuscript fully available?

The [PLOS Data policy](http://www.plosone.org/static/policies.action#sharing) requires authors to make all data underlying the findings described in their manuscript fully available without restriction, with rare exception (please refer to the Data Availability Statement in the manuscript PDF file). The data should be provided as part of the manuscript or its supporting information, or deposited to a public repository. For example, in addition to summary statistics, the data points behind means, medians and variance measures should be available. If there are restrictions on publicly sharing data—e.g. participant privacy or use of data from a third party—those must be specified.

The dataset of this study is available in the Supporting Information (S2 dataset.sav)

Reviewer #1: Yes

4. Is the manuscript presented in an intelligible fashion and written in standard English?

PLOS ONE does not copyedit accepted manuscripts, so the language in submitted articles must be clear, correct, and unambiguous. Any typographical or grammatical errors should be corrected at revision, so please note any specific errors here.

Reviewer #1: Yes

5. Review Comments to the Author

Please use the space provided to explain your answers to the questions above. You may also include additional comments for the author, including concerns about dual publication, research ethics, or publication ethics. (Please upload your review as an attachment if it exceeds 20,000 characters)

Reviewer #1: This manuscript by Rang et al addresses an important theme of the effect of secondhand smoking on preterm delivery. There have been several comprehensive articles addressing a parallel theme that reported higher incidence of preterm birth in women exposed to secondhand smoking. The authors of this manuscript confirm earlier findings and suggest that this happens to be true in Vietnamese population. The study uses a sizeable cohort but lacks some rigorous protocols that should have been used and described to strengthen the study.
Major comments:
1. The authors should use some of the rigorous tools that have been described in a similar study by Ashford et al (The effects of prenatal secondhand smoke exposure on preterm birth and neonatal outcomes. J Obstet Neonatal Nurs. 2010; 39:525-535)..

Measuring nicotine in maternal hair to confirm exposure to secondhand smoking as the study of Ashford *et al.* may be more reliable than interviewing tobacco exposures by members smoking at home; however, this test is not available in Vietnam due to the cost. According to Perez-Rios M *et al.* (2013), 84% (309/369) of the secondhand smoking-related studies have used interview questionnaires so far; only 16 % (50/369) of the studies measured cotinine/ nicotine in hair, urine or saliva.

The advantage of questionnaires is that they allow for a detailed ascertainment of exposure and this should be specially valued. Questionnaires also obtain information regarding the intensity and duration of the smoke exposure at home.

In the future, we will design a study to compare the nicotine concentration in hair with the number of cigarettes per day smoked at home.

Pérez-Ríos M et al. Questionnaire-based second-hand smoke assessment in adults. Eur J Public Health. 2013;23(5):763-7.

2. How did the authors confirm exposure to secondhand smoking? Was it solely based on questionnaire or was a maternal hair nicotine estimation performed on subjects and controls? Such a statement or description is not provided. Without such a quantification of maternal hair nicotine, it is hard to base the authors’ conclusions on verbal or written answers.

The exposure to the secondhand smoking is this study is based on the validated questionnaires after conducting the pilot study with sample size of 40 women at the Obstetrics ward. This SHS questionnaires are available in the Supporting Information of the revised manuscript

3. Were the pregnant women asked about the exposure to smoking prior to or after delivery? It is possible that the women looked for a reason for their preterm deliveries if they were asked after delivery.

This is a case-control study, so the interviews were conducted after delivery when the outcome (preterm birth, full-term birth) have already occurred. All women in the case and control group were asked the exposure to the secondhand smoking. The disadvantages of the case-control study were presented in the limitations of the study in the manuscript

4. Did the authors compare the incidence of preterm birth between women with active smoking and women with secondhand exposure? This is very important because this has not been studied.

Nearly all Vietnamese women don’t smoke (1%), moreover the women who smokes were excluded from the present study. Therefore, we don’t have the data related to women with active smoking. All the pregnant women in this study were secondhand smoking

5. Results: If the reviewer understands it clearly, 67.4% women with preterm birth revealed that they were exposed to secondhand smoke compared to 51% with term pregnancy. This means 51% women with term pregnancy also were exposed. Since 51% exposed women experienced term pregnancy, there must be a second confounding factor in women with preterm birth. Can the authors elaborate on this issue?

In a case-control study, as the outcome occurred (preterm of term), interviewers will ask about the past exposures (secondhand smoking), of course, the exposure was found in both groups, but the proportion of exposure was different between the two groups (67.5% in preterm vs. 51% in full-term group). From the difference we calculate the odds ratio. In this study OR1 for being preterm was 194/94 and OR2 for being term baby was 147/141, and crude OR will be: OR1/OR2= 194/94: 147/141=1.97. The secondhand smoking is the main predictor, not a confounding factor in this study.

6. PLOS authors have the option to publish the peer review history of their article ([what does this mean?](https://journals.plos.org/plosone/s/editorial-and-peer-review-process#loc-peer-review-history)). If published, this will include your full peer review and any attached files.

If you choose “no”, your identity will remain anonymous but your review may still be made public.

No

**Do you want your identity to be public for this peer review?** For information about this choice, including consent withdrawal, please see our [Privacy Policy](https://www.plos.org/privacy-policy).

Reviewer #1: No

[NOTE: If reviewer comments were submitted as an attachment file, they will be attached to this email and accessible via the submission site. Please log into your account, locate the manuscript record, and check for the action link "View Attachments". If this link does not appear, there are no attachment files.]

While revising your submission, please upload your figure files to the Preflight Analysis and Conversion Engine (PACE) digital diagnostic tool, <https://pacev2.apexcovantage.com/>. PACE helps ensure that figures meet PLOS requirements. To use PACE, you must first register as a user. Registration is free. Then, login and navigate to the UPLOAD tab, where you will find detailed instructions on how to use the tool. If you encounter any issues or have any questions when using PACE, please email PLOS at [figures@plos.org](mailto:figures@plos.org). Please note that Supporting Information files do not need this step.
